# Fragmentation in adolescent health care provision

**DOI:** 10.1192/bjo.2025.10059

**Published:** 2025-06-08

**Authors:** Mina Fazel, Emma Soneson

**Affiliations:** Department of Psychiatry, University of Oxford, UK; Children’s Psychological Medicine, Oxford Children’s Hospital, Oxford University Hospitals NHS Foundation, Oxford, UK

**Keywords:** Adolescent mental health, service transition, integrated care models, in-patient psychiatry, mental health service design

## Abstract

This editorial argues for integrated, developmentally informed models of mental health care for adolescents that address pervasive structural misalignments across health, education and social care. Adolescent admissions must be understood within a whole-system and lifespan framework, recognising varied reasons for admission and long-term impacts on engagement, trust and identity.

**Figure:**
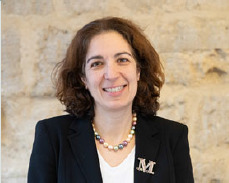

A crisis often acts as a catalyst for change, exposing service gaps that might otherwise remain obscured. This is particularly true in the case of adolescent care (defined here as those 10–24 years of age^1^), where current pressures, which have been increasing year on year, demand urgent reconsideration in terms of how best to support young people with serious and complex health needs. The paper, published in this issue by Burn et al^2^ on the perceived impacts of young people’s admissions to adult mental health wards, illuminates just one dimension of a broader, systemic issue – how to provide safe, developmentally appropriate and therapeutic care for adolescents within a fragmented service landscape.^3^

The institutions at the heart of statutory provision for adolescents – health, social care and education – operate under different legal and organisational frameworks, even on some of the most fundamental matters. One key point of divergence concerns the age at which a young person is deemed an adult, and thus literally treated alongside adults. In UK healthcare, this manifests starkly: paediatric services typically end at age 16, while Child and Adolescent Mental Health Services (CAMHS) extend to age 18.^4^ Similarly, education is compulsory until age 16 and, although most statutory support in social care ceases at age 18, care leavers can draw on extended support until the age of 25.

These diverse and fairly arbitrary age limits lead to the formation of a group of young people who are, as one participant in the paper by Burn et al aptly described, ‘in that funny in-between age’.^2^ As they approach the ‘transition boundary’,^5^ these young people often slip through the cracks created by a range of financial, organisational and procedural barriers that disrupt care, resulting in suboptimal transition into adult services and negative experiences on behalf of the young person and their family that may lead them to disengage from services altogether.^5–7^ For many adolescents, these transitions in care play out against a backdrop of other major life transitions,^8^ which may further complicate the picture for a group where continuity of care may be especially important.

Even within a single service, the transition at age 18 raises practical and ethical questions: does care automatically transfer to a new team on a specific date when little else is likely to be changing for that young person in terms of their cognitive, emotional and social needs? Must a young person admitted to one ward be relocated simply due to age? Is continuity of care maintained or disrupted by administrative boundaries? For those with multifaceted health and care needs, these questions become increasingly relevant, as the differences in transition age across settings and services may disrupt the interconnected ‘networks of care’^9^ that have formed around the young person.^8^

It is essential to distinguish the different purposes behind the admission of an adolescent with mental health needs before assessing the impact of the admission’s setting. These purposes can be grouped into several, often overlapping, thematic categories. The most immediate is crisis stabilisation, frequently accompanied by risk management and safeguarding concerns. Other clinical drivers include the need for either specialist assessment or intensive treatment and monitoring, particularly when out-patient and community-based resources are unavailable or insufficient. In addition to these medical reasons, admissions may occur for non-clinical purposes, such as containment following the breakdown of a care placement.

Nevertheless, the function of admission must be considered alongside its developmental impact. These admissions do not exist in isolation: their effects reverberate well beyond any immediate crisis. An adolescent’s admission to hospital carries long-term implications for their trajectory of care and even self-perception. Hospitalisation during this formative stage often shapes a young person’s understanding of their condition,^10^ and of the health system more broadly. For instance, a 17-year-old admitted with chronic liver disease may find themselves placed on a ward alongside octogenarians – a placement that is developmentally incongruent and emblematic of a wider issue across all of healthcare, not just within mental health. Early and developmentally appropriate interventions have been shown to improve long-term outcomes – including treatment adherence, self-management of chronic conditions and (mental) health literacy. Conversely, if a young person’s first experience of intensive care is in an ill-fitting environment – be it adult-centred, risk-focused or overly restrictive – it can have lasting negative effects, particularly on their willingness to engage with services during future episodes of illness, prompting concern that has fuelled investigation into alternative models of care delivery to reduce or avoid the need for in-patient admission.^11,12^

Equally important are the relational dynamics within therapeutic care, especially for adolescents in the context of hospitalisation. Adolescents place heightened value on peer relationships and appreciate the opportunity to connect with others facing similar challenges; these peer connections often serve therapeutic functions in themselves, fostering belonging, shared understanding and emotional validation. Nevertheless, institutional arrangements that separate adolescents from age-matched peers – whether due to arbitrary age thresholds, bed availability or administrative silos – can disrupt these informal but crucial supports. For many, peer connection and mutual support are as central to recovery as medication or formal therapy. Denying these opportunities through fragmented systems not only impedes individual healing but also undermines foundational principles of in-patient psychiatric treatment and therapeutic milieu.^13^

In considering how best to serve young people with mental illness, what is needed is not a rigid adherence to an 18th birthday as a marker of maturity, but rather a recognition that adolescence – by its very nature – is transitional. This transition extends beyond developmental milestones and cuts across institutional boundaries in health, education and social care. Coordinated, developmentally appropriate provision must acknowledge that administrative thresholds do not necessarily align with cognitive, emotional or social readiness. The example of a 17-year-old with chronic health difficulties placed on an adult ward exemplifies a much broader issue. It is not simply a matter of mental health care failing to adapt – this is a challenge across many domains of adolescent care.

What is urgently required is integrated, developmentally attuned provision for adolescents – for example, spanning the ages of 14 to 24 years – that bridges the arbitrary divides between acute and community care, between somatic (physical) and mental healthcare and between child and adult systems.^7,14,15^ Such models need to recognise the holistic needs of this population, not only to support crisis recovery but also to sustain long-term engagement. Across the globe, innovative models of adolescent health care are emerging and, while these are promising, their careful evaluation will be essential to understanding their effectiveness and applicability in different contexts.^15,16^

What we cannot afford – especially when existing systems are so clearly not designed with Gen Z in mind – is a failure to innovate. This is a generation more digitally connected and globally aware than any before it, accustomed to systems that are fast, intuitive and responsive. However, when they enter the health system, particularly in crisis, they encounter structural inconsistencies, misaligned thresholds and fragmented provision. They may be ‘in that funny in-between age’, but there is nothing amusing about the consequences.
